# Clinical Perspective of FDA Approved Drugs With P-Glycoprotein Inhibition Activities for Potential Cancer Therapeutics

**DOI:** 10.3389/fonc.2020.561936

**Published:** 2020-11-16

**Authors:** Jiun-I Lai, Yu-Jhen Tseng, Ming-Huang Chen, Chi-Ying F. Huang, Peter Mu-Hsin Chang

**Affiliations:** ^1^ Division of Medical Oncology, Department of Oncology, Taipei Veterans General Hospital, Taipei, Taiwan; ^2^ Center for Immuno-Oncology, Department of Oncology, Taipei Veterans General Hospital, Taipei, Taiwan; ^3^ Institute of Clinical Medicine, School of Medicine, National Yang-Ming University, Taipei, Taiwan; ^4^ School of Medicine, National Yang-Ming University, Taipei, Taiwan; ^5^ Institute of Biopharmaceutical Sciences, National Yang-Ming University, Taipei, Taiwan

**Keywords:** p-glycoprotein, repurposing, cancer, drug resistance, chemotherapy

## Abstract

P-glycoprotein (also known as multidrug resistance protein 1 (MDR1) or ATP-binding cassette sub-family B member 1 (*ABCB1*) plays a crucial role in determining response against medications, including cancer therapeutics. It is now well established that p-glycoprotein acts as an ATP dependent pump that pumps out small molecules from cells. Ample evidence exist that show p-glycoprotein expression levels correlate with drug efficacy, which suggests the rationale for developing p-glycoprotein inhibitors for treatment against cancer. Preclinical and clinical studies have investigated this possibility, but mostly were limited by substantial toxicities. Repurposing FDA-approved drugs that have p-glycoprotein inhibition activities is therefore a potential alternative approach. In this review, we searched the Drugbank Database (https://www.drugbank.ca/drugs) and identified 98 FDA-approved small molecules that possess p-glycoprotein inhibition properties. Focusing on the small molecules approved with indications against non-cancer diseases, we query the scientific literature for studies that specifically investigate these therapeutics as cancer treatment. In light of this analysis, potential development opportunities will then be thoroughly investigated for future efforts in repositioning of non-cancer p-glycoprotein inhibitors in single use or in combination therapy for clinical oncology treatment.

## Introduction

Chemotherapy has been the mainstay of cancer treatment for several decades, and is still an indispensable treatment component in current standard of care. The ability of cancer cells to acquire resistance against cytotoxic chemotherapy is one of the basis for cancer progression. One major mechanism for chemotherapy resistance formation is drug efflux by the ATP-binding cassette (ABC) transporter family of transmembrane proteins (1). These proteins share a common ability to pump chemotherapy agents across the plasma membrane with no prerequisite for structure similarity. The discovery of outward efflux of daunorubicin led to the first report of chemotherapy resistance mechanism by an ABC transporter family protein in 1973 (2). Further work demonstrated that this protein was a cell surface glycoprotein that was later on named P-glycoprotein (p-gp) (3). In the upcoming years, several other proteins of the ABC transporter family were identified that shared a common mechanism of conferring chemotherapy resistance by drug efflux (4). However, p-gp remains the best studied and most potent ABC transporter to induce chemoresistance. The drugs that are amenable to p-gp transport includes many of the most commonly used chemotherapy agents such as anthracyclines, taxenes, vinca alkaloids, and many others (5). In the human body, p-gp is widely expressed in a variety of organs including the liver, kidney and gastrointestinal tract (6), and upregulation of p-gp is closely linked with increased chemoresistance in cancer (7).

## Early Development Efforts of First Generation p-gp Inhibitors in Cancer Treatment

With strong mechanistic evidence for p-gp in mediating chemoresistance, a logical therapeutic approach would be to utilize p-gp inhibitors against cancers, with the rationale of enhancing chemotherapy efficacy. Indeed, in the past decades, many efforts have been made to incorporate p-gp inhibitors in clinical trials for cancer patients. Earliest efforts of p-gp inhibitors in cancer included trials using verapamil (8) and cyclosporine (9) but they were unsuccessful and demonstrated many adverse effects (10). Verapamil is a phenylalkylamine that blocks voltage-dependent L-type calcium channels and is used for treatment of hypertension. In a randomized phase II clinical trial in patients diagnosed with small cell lung cancer receiving cyclophosphamide, doxorubicin, vincristine, and etoposide (CAVE), a daily dose of 480 mg was given in combination with chemotherapy, with hypotension as a dose limiting toxicity for verapamil (11). Even at this high dosage, clinical trials failed to demonstrate a benefit for verapamil (11, 12). Further analysis demonstrated that blood levels of verapamil was significantly lower than the dosage proven efficacious in preclinical studies (11), despite already nearing maximum tolerated doses (MTD). Verapamil is representative of the so called “first generation” p-gp inhibitors that utilize drugs with known mechanism of action (MOA). These agents include cyclosporine (13, 14), quinidine (15), tamoxifen (16), and others. In summary, first generation p-gp inhibitors suffer from low therapeutic window, poor efficacy in combination with chemotherapy, and generally low potential for further development (10).

## Second Generation p-gp Inhibitors

Second generation p-gp inhibitors aimed to improve the therapeutic window of first generation agents. Dexverapamil is the R-form of verapamil with reduced potency in inhibiting p-gp but also with reduced cardiotoxicity. In a phase I/II trial (17), dexverapamil was combined with vinblastine in treatment of renal cell carcinoma. The toxicity profile was tolerable in comparison to trials using verapamil, with the majority being mild or asymptomatic cardiac related side effects. No partial or complete responses were seen, which is not unexpected given the poor chemosensitivity of renal cell carcinoma. A phase II study tested the hypothesis of dexverapamil in reversing chemoresistance, by adding dexverapamil to cancer patients who had disease progression on anthracyclines (18). Among 21 patients, 2 had partial responses and 2 had stable disease, with a disease control rate (DCR) of 19%, demonstrating a modest activity. It should be highlighted that this trial used the same anthracycline backbone and added dexverapamil upon documented progression, therefore demonstrating the anticipated drug resistance reversal abilities of p-gp inhibition.

S9788 is another p-gp inhibitor that showed MDR reversal in a phase I trial in combination with doxorubicin in colorectal cancer and renal cell carcinoma patients, although cardiac arrhythmia was a major dose limiting toxicity (19). In another phase I trial, also in combination with doxorubicin, arrhythmia including AV-block, QT prolongation, ventricular arrhythmia and torsade de pointes precluded further development of the trial and essentially for the drug altogether (20).

PSC-833, an analog of cyclosporine with a significantly improved toxicity profile and enhanced p-gp inhibitory ability (21, 22), underwent several clinical trials for development as a potent second generation agent. In phase I trials, PSC-833 demonstrated acceptable toxicity profiles (23), and was further developed in combination with chemotherapy, mainly in leukemia patients with the aim to delay ensuing drug resistance from first line MEC (mitoxantrone, etoposide, ara-C) induction chemotherapy (24–26). Some degree of success was observed in these earlier phase trials, leading to several phase III trials utilizing PSC-833 in leukemia (27) and solid tumor or multiple myeloma (28, 29). However, no benefit was seen in any of these phase III trials with addition of PSC-833, dampening the initial excitement and expectations for this drug.

## Third Generation p-gp Inhibitors

With the unsuccessful attempts in clinical development for p-gp inhibitors, medicinal chemistry work developed newer agents with high selectivity and efficacy, including zosuquidar, elaquidar, laniquidar (R101933), and tariquidar (XR9576) (30). Although currently no p-gp inhibitors have been approved for cancer treatment, the clinical development of these novel agents are worth mentioning in detail, which may lead to further understanding of how to utilize MDR reversal in oncology therapy. In the following sections, we discuss the properties of each agent and their clinical development, with a focus on future perspectives for further development.

## Zosuquidar

Zosuquidar trihydrochlorid, previously known as LY335979, was developed as a potent and selective inhibitor against p-gp, with minimal inhibitory effect on other MDR proteins (MRP1, MRP2) (31). A phase I trial investigated oral zosuquidar alone and in combination with doxorubicin in advanced malignancies to determine its safety profile (32). Dose limiting toxicities included cerebellar dysfunction, hallucinations, and palinopsia, but overall the drug was well tolerated. Subsequently, another phase I trial investigated the intravenous form of zosuquidar in combination with doxorubicin in cancer (33). In this trial, neurotoxicity was markedly less severe and less frequent, suggesting a different pharmacokinetic profile between oral and intravenous formulation of zosuquidar. It must be noted that although phase I trials were not designed for efficacy, further analysis for these 2 trials did not demonstrate a striking improvement in response. A phase II trial investigated the addition of zosuquidar to docetaxel in metastatic breast cancer. Although the combination was safe, the conclusion of the trial did not demonstrate any difference in progression free survival (PFS), overall survival (OS), or objective response rate (ORR) in patients with metastatic breast cancer.

Zosuquidar was also investigated in the setting of hematological cancers. In a phase I trial with acute leukemia patients, zosuquidar was combined with standard induction chemotherapy using daunorubicin and cytarabine (34). In a small sample size of 16 patients, grade 3/4 adverse effects including respiratory failure, hypokalemia, arrhythmia, and febrile neutropenia were present. Seven patients achieved a complete response (CR), with a CR rate of 43.7%. Considering that standard induction chemotherapy in AML already has a well-established ORR of 50–60% (35), it is difficult to conclude that zosuquidar provided significant additional benefit (although it must be highlighted that this is a relatively small study). In further analysis, 2 out of 4 patients with p-gp negative cancers (according to IHC staining) had CR (50%) while 5 out of the 12 patients with p-gp positive cancers had CR (41.6%), further suggesting that zosuquidar might not provide anticipated efficacy when added to standard induction chemotherapy to AML (acute myeloid leukemia). A confirmatory phase III trial was performed in elderly (over 60 years old) AML patients, receiving standard cytarabine and daunorubicin in addition to either zosuquidar or placebo (36). The response rate and median 2-year OS were similar (20 vs 23%, 7.2 months vs 9.4 months, respectively for with versus without zosuquidar). In an accompanying editorial, the authors rechallenged the validity of using p-gp as the target for leukemia based on this trial and other accumulated trial results (37). They suggest that although p-gp and other MDR proteins including MRP, LRP, and BRCP were indeed expressed by leukemia cells in the majority of patients in the trial, there was no correlation between the expression status of MDR proteins and survival (37). This editorial came to the conclusion that further MDR inhibition in the setting of AML should be highly reconsidered in light of the result from these well designed clinical trials.

## Elacridar

Elacridar is a potent and specific inhibitor of p-gp that works by modulating the ATPase activity (38). The efficacy of elacridar in inhibiting p-gp and its effect in plasma concentrations of other drugs have been extensively characterized in multiple studies [for a comprehensive review, please refer to (39)]. Phase I studies for elacridar demonstrated very minor side effects and good pharmacokinetic properties (40). In another early phase trial, elacridar was shown to increase the plasma levels of oral paclitaxel (41), by inhibiting intestinal p-gp activity. Interestingly, when these patients were switched to intravenous paclitaxel in subsequent cycles, the area under curve (AUC) of plasma paclitaxel when using oral paclitaxel plus elacridar was still significantly lower compared to intravenous paclitaxel (41). This suggested a modest effect of elacridar to augment oral paclitaxel levels in patients. Similarly, in another early phase trial, elacridar significantly elevated the oral bioavailability of topotecan to more than 2 fold when used in combination with oral topotecan (42), although blood topotecan levels was still lower than the levels of intravenous topotecan. Nonetheless, this result implicated a potential application for elacridar to be combined with oral chemotherapy for long-term use. Similar conclusions were made in another phase I trial (43). When combining with intravenous chemotherapy Elacridar was demonstrated to be safe with relatively mild side effects in combination with doxorubicin in solid cancers (44). The most common toxicity was neutropenia. Despite the acceptable safety profile of these early phase trials, elacridar was not further developed in later clinical trials.

## Tariquidar

Tariquidar is a potent and specific inhibitor of p-gp, acting as an ATPase inhibitor (45). After demonstrating preclinical efficacy, early clinical trials demonstrated good tolerability with minimal side effects in healthy individuals (46). A phase I study combining tariquidar and vinorelbine demonstrated good tolerability, and more importantly, the lack of pharmacokinetic interactions between tariquidar and vinorelbine, as this was found to be a common issue with p-gp inhibitors in human trials (47). However, there was overall a lack of response in this trial. In a phase II study tariquidar was added to the standard chemotherapy (anthracyclines or taxanes) in breast cancer patients’ refractory on these regimens. Tariquidar was added to the current regimen to observe if drug resistance could be reversed. A total of 17 patients were enrolled, and only one patient demonstrated a partial response (ORR: 6%). 5 out of the 17 patients had a positive p-gp staining before treatment, including the patient with response. Therefore, it could be estimated that in p-gp positive patients, the response rate was 20%, albeit a very small sample size (n=5). Interestingly, this trial measured the uptake of technetium-99m (^99m^Tc) as a surrogate for whether the anticipated biology effect of improving drug influx is seen. The strongest increase of ^99m^Tc occurred in the single patient with the response. This suggested that ^99m^Tc might be a viable indicator of patients that could benefit from tariquidar and other p-gp inhibitors. This phenomenon led to an unexpected development of tariquidar into a potential radiotracer when coupled with radioactive [11C] labeling (48). This has become an active area of research for tariquidar and other p-gp inhibitors (49–51). Another phase I trial combined tariquidar with docetaxel in patients with lung, ovarian and cervical cancer (52). This trial confirmed that tariquidar was associated with increased ^99m^Tc sestamibi uptake, however this finding did not translate into a significant increase in ORR as only 10% of patients had a response in this trial. A more recent phase I trial combined tariquidar with doxorubicin, vinorelbine, or docetaxel in children and adolescents with refractory solid tumors (53). An ORR of approximately 10% was observed. Overall, tariquidar clinical trials were similar to other third generation p-gp inhibitors, demonstrating relatively tolerable safety profiles, but with unimpressive anticancer efficacy. We summarize the pg-inhibitors in **Table 1**.

**Table 1:** FDA approved drugs with p-gp inhibition activities.

DrugBank ID	Name	MOA (mechanism of action)	Recruitment status	Cancer type	Combined treatment	Phase	Reference
DB00678	Losartan	Angiotensin II receptor antagonist	Recruiting	Pancreatic cancer	Nivolumab	Phase II	https://clinicaltrials.gov/ct2/show/NCT03563248
DB00970	Dactinomycin	Antibacterial	Completed	Gestational Trophoblastic Tumor	N/A	Phase II	https://clinicaltrials.gov/ct2/show/NCT00003688
			Completed	Extragonadal Germ Cell Tumor Ovarian Cancer	Chemotherapy	Phase II	https://clinicaltrials.gov/ct2/show/NCT00002489
DB01137	Levofloxacin	Antibacterial	Completed	Solid Tumors or Lymphoma	N/A	Phase III	https://clinicaltrials.gov/ct2/show/NCT00005590
DB00027	Gramicidin D	Antibacterial	N/A	N/A	N/A	N/A	N/A
DB00365	Grepafloxacin	Antibacterial	N/A	N/A	N/A	N/A	N/A
DB11753	Rifamycin	Antibacterial	N/A	N/A	N/A	N/A	N/A
DB00555	Lamotrigine	Antiepileptic	Completed	Unspecified Adult Solid Tumor	N/A	Phase III	https://clinicaltrials.gov/ct2/show/NCT00068445
DB01263	Posaconazole (Multiple)	Antifungal	Completed	Hematologic Malignancies	N/A	Phase III	https://clinicaltrials.gov/ct2/show/NCT00750737
			Completed	Solid Tumor	Idasanutlin	Phase I	https://clinicaltrials.gov/ct2/show/NCT01901172
			Completed	Leukemia	N/A	Phase II	https://clinicaltrials.gov/ct2/show/NCT00936117
DB00341	Cetirizine	Antihistamine	Recruiting	Oncology Patients Receiving Chemotherapy	N/A	Phase II	https://clinicaltrials.gov/ct2/show/NCT04189588
			Recruiting	Solid Tumor	N/A	Phase III	https://clinicaltrials.gov/ct2/show/NCT04237090
DB00863	Ranitidine	Antihistamine	Active, not recruiting	Cancers	N/A	Phase IV	https://clinicaltrials.gov/ct2/show/NCT03145012
			Completed	Medullary Thyroid Cancer	N/A	Phase I	https://clinicaltrials.gov/ct2/show/NCT01539655
DB00455	Loratadine	Antihistamine	N/A	N/A	N/A	N/A	N/A
DB00243	Ranolazine	Anti-ischemia	Completed	Prostate Cancer	N/A	N/A	https://clinicaltrials.gov/ct2/show/NCT01992016
DB00358	Mefloquine	Antiparasite	Active, not recruiting	Glioblastoma	N/A	Phase I	https://clinicaltrials.gov/ct2/show/NCT01430351
DB00468	Quinine	Antiparasite	N/A	N/A	N/A	N/A	N/A
DB00608	Chloroquine	Antiparasite	Terminated	Small Cell Lung Cancer	N/A	Phase I	https://clinicaltrials.gov/ct2/show/NCT01575782
			Unknown	Breast Cancer	N/A	Phase II	https://clinicaltrials.gov/ct2/show/NCT02333890
			Completed	Solid Tumor	N/A	Phase I	https://clinicaltrials.gov/ct2/show/NCT02071537
DB00975	Dipyridamole	Antiplatelet	Unknown	Ovarian Cancer	methotrexate	Phase II	https://clinicaltrials.gov/ct2/show/NCT00002487
DB08816	Ticagrelor	Antiplatelet	N/A	N/A	N/A	N/A	N/A
DB00316	Acetaminophen	Antipyretic	Completed	Chronic Myeloid Leukemia (CML)	Imatinib	Phase I	https://clinicaltrials.gov/ct2/show/NCT00428909
DB11586	Asunaprevir	Antiviral	N/A	N/A	N/A	N/A	N/A
DB05521	Telaprevir	Antiviral	N/A	N/A	N/A	N/A	N/A
DB06290	Simeprevir	Antiviral	Unknown	Hepatocellular Carcinoma	N/A	Phase III	https://clinicaltrials.gov/ct2/show/NCT02771405
DB09102	Daclatasvir	Antiviral	N/A	N/A	N/A	N/A	N/A
DB11574	Elbasvir	Antiviral	Active, not recruiting	Advanced Refractory Liver Cancer	N/A	Phase I/II	https://clinicaltrials.gov/ct2/show/NCT02940496
DB11613	Velpatasvir	Antiviral	Active, not recruiting	Hepatitis C virus-associated indolent B-cell lymphoma	Sofosbuvir	Phase II	https://clinicaltrials.gov/ct2/show/NCT02836925
DB12026	Voxilaprevir	Antiviral	N/A	N/A	N/A	N/A	N/A
DB13878	Pibrentasvir	Antiviral	N/A	N/A	N/A	N/A	N/A
DB13879	Glecaprevir	Antiviral	N/A	N/A	N/A	N/A	N/A
DB12070	Letermovir	Antiviral	N/A	N/A	N/A	N/A	N/A
DB00300	Tenofovir disoproxil	Antiviral	Active, not recruiting	Hepatocellular Carcinoma	N/A	N/A	https://clinicaltrials.gov/ct2/show/NCT02129829
DB08893	Mirabegron	Beta agonist	Completed	Myeloproliferative Neoplasm	N/A	Phase II	https://clinicaltrials.gov/ct2/show/NCT02311569
DB00612	Bisoprolol	Beta blockers	Recruiting	Breast Cancer	N/A	Phase III	https://clinicaltrials.gov/ct2/show/NCT02236806
DB00661	Verapamil	Calcium channel blocker	Completed	Brain Cancer Meningioma	Hydroxyurea	Phase II	https://clinicaltrials.gov/ct2/show/NCT00706810
DB01388	Mibefradil	Calcium channel blocker	Completed	Brain and Central Nervous System Tumor	Temozolomide	Phase I	https://clinicaltrials.gov/ct2/show/NCT01480050
DB00622	Nicardipine	Calcium channel blocker	Completed	Brain Tumor	N/A	Phase I	https://clinicaltrials.gov/ct2/show/NCT01951950
DB00381	Amlodipine	Calcium channel blocker	Recruiting	Metastatic Triple Negative Breast Cancer	N/A	Phase I/II	https://clinicaltrials.gov/ct2/show/NCT02834403
DB00343	Diltiazem	Calcium channel blocker	N/A	N/A	N/A	N/A	N/A
DB11712	Tezacaftor	CFTR drug	N/A	N/A	N/A	N/A	N/A
DB15444	Elexacaftor	CFTR drug	N/A	N/A	N/A	N/A	N/A
DB04348	Taurocholic acid	Cholerectic	N/A	N/A	N/A	N/A	N/A
DB01200	Bromocriptine	Dopamine agonist	N/A	N/A	N/A	N/A	N/A
DB00477	Chlorpromazine	Dopamine antagonist	Active, not recruiting	Advanced, Metastatic or Recurrent Cancer	Haloperidol	Phase II/III	https://clinicaltrials.gov/ct2/show/NCT03021486
DB00502	Haloperidol	Dopamine antagonist	Completed	Advanced Cancer	N/A	Not Applicable	https://clinicaltrials.gov/ct2/show/NCT01539733
			Recruiting	Advanced Cancer	Lorazepam	Phase II/III	https://clinicaltrials.gov/ct2/show/NCT03743649
DB01267	Paliperidone	Dopamine inhibitor	N/A	N/A	N/A	N/A	N/A
DB11979	Elagolix	GnRH inhibitor	N/A	N/A	N/A	N/A	N/A
DB13874	Enasidenib	IDH inhibtor	Not yet recruiting	Acute Myeloid Leukemia	Venetoclax	Phase I/II	https://clinicaltrials.gov/ct2/show/NCT04092179
			Not yet recruiting	Accelerated/Blast-phase Myeloproliferative Neoplasm Chronic-phase Myelofibrosis	Ruxolitinib	Phase II	https://clinicaltrials.gov/ct2/show/NCT04281498
			Completed	Solid Tumor Glioma Angioimmunoblastic T-cell Lymphoma Intrahepatic Cholangiocarcinoma Chondrosarcoma	N/A	Phase I/II	https://clinicaltrials.gov/ct2/show/NCT02273739
DB14568	Ivosidenib	IDH inhibtor	Not yet recruiting	Advanced Solid Tumor	Nivolumab	Phase II	https://clinicaltrials.gov/ct2/show/NCT04056910
			Recruiting	Advanced Hematologic Malignancies	N/A	Phase I	https://clinicaltrials.gov/ct2/show/NCT02074839
			Recruiting	IDH1 Mutant Chondrosarcoma	N/A	Phase II	https://clinicaltrials.gov/ct2/show/NCT04278781
DB00091	Cyclosporine	Interleukin inhibitor	Completed	Metastatic Breast Cancer	Nab-paclitaxel	Phase I	https://clinicaltrials.gov/ct2/show/NCT00983424
			Completed	Colorectal Cancer	CPT-11	Phase II	https://clinicaltrials.gov/ct2/show/NCT00003950
			Completed	Cervical Cancer Vaginal Cancer	N/A	Phase II	https://clinicaltrials.gov/ct2/show/NCT00005941
			Completed	Leukemia	N/A	Phase II	https://clinicaltrials.gov/ct2/show/NCT00185640
			Completed	Brain Tumors Central Nervous System Tumors	N/A	Phase I	https://clinicaltrials.gov/ct2/show/NCT00003625
DB00199	Erythromycin	Macrolide	N/A	N/A	N/A	N/A	N/A
DB01211	Clarithromycin	Macrolide	Unknown	Squamous Cell Lung Cancer Non-Squamous Cell Lung Cancer Non-Small Cell Lung Cancer	Treosulfan pioglitazone	Phase II	https://clinicaltrials.gov/ct2/show/NCT02852083
			Completed	Neoplasm	Abemaciclib	Phase I	https://clinicaltrials.gov/ct2/show/NCT02117648
			Completed	Lymphoma	N/A	N/A	https://clinicaltrials.gov/ct2/show/NCT00461084
			Recruiting	Multiple Myeloma	Thalidomide Cyclophosphamide Dexamethasone	Phase III	https://clinicaltrials.gov/ct2/show/NCT02248428
DB00207	Azithromycin	Macrolide	N/A	N/A	N/A	N/A	N/A
DB00328	Indomethacin	NSAID	Recruiting	Recurrent or Metastatic Hormone-Resistant Prostate Cancer	N/A	Phase I/II	https://clinicaltrials.gov/ct2/show/NCT02935205
			Completed	Colorectal Cancer	N/A	Phase IV	https://clinicaltrials.gov/ct2/show/NCT00473980
			Active, not recruiting	Early Stage Breast Cancer Triple Negative Breast Cancer	N/A	Phase I	https://clinicaltrials.gov/ct2/show/NCT02950259
DB00203	Sildenafil	PDE5 inhibitor	Completed	Non-small Cell Lung Cancer	Carboplatin/Paclitaxel	Phase II/III	https://clinicaltrials.gov/ct2/show/NCT00752115
			Completed	Solid Cancer	Regorafenib	Phase I	https://clinicaltrials.gov/ct2/show/NCT02466802
			Completed	Kidney Cancer	N/A	Phase I	https://clinicaltrials.gov/ct2/show/NCT01950923
			Completed	Waldenstrom’s Macroglobulinemia	N/A	Phase II	https://clinicaltrials.gov/ct2/show/NCT00165295
DB00862	Vardenafil	PDE5 inhibitor	Terminated	Glioma Brain Metastasis	Carboplatin	Early Phase I	https://clinicaltrials.gov/ct2/show/NCT02279992
DB14057	Valinomycin	Potassium channel transporter	N/A	N/A	N/A	N/A	N/A
DB00736	Esomeprazole	Proton pump inhibitors	Completed	Metastatic ErbB2 Positive Breast Cancer	Lapatinib	Phase I	https://clinicaltrials.gov/ct2/show/NCT00849329
			Completed	Esophageal Cancer	N/A	Phase II	https://clinicaltrials.gov/ct2/show/NCT00474903
			Completed	Colorectal Cancer	N/A	Phase IV	https://clinicaltrials.gov/ct2/show/NCT00473980
DB00213	Pantoprazole	Proton pump inhibitors	Active, not recruiting	Prostate Cancer	N/A	Phase II	https://clinicaltrials.gov/ct2/show/NCT01748500
			Completed	Advanced Solid Tumor	Doxorubicin	Phase I	https://clinicaltrials.gov/ct2/show/NCT01163903
DB00338	Omeprazole	Proton pump inhibitors	Not yet recruiting	Prostate Cancer	N/A	Phase II	https://clinicaltrials.gov/ct2/show/NCT04337580
			Completed	Colorectal Cancer	N/A	Phase II	https://clinicaltrials.gov/ct2/show/NCT02518373
			Completed	Solid Tumor	Patupilone	Phase I	https://clinicaltrials.gov/ct2/show/NCT00420615
DB00448	Lansoprazole	Proton pump inhibitors	Not yet recruiting	Breast Cancer	Avelumab	Phase II	https://clinicaltrials.gov/ct2/show/NCT04188119
DB11732	Lasmiditan	Serotonin receptor agonist	N/A	N/A	N/A	N/A	N/A
DB08907	Canagliflozin	SGLT-2 inhibitor	Not yet recruiting	Advanced Solid Tumors	N/A	Phase I/II	https://clinicaltrials.gov/ct2/show/NCT04073680
DB00285	Venlafaxine	SNRI	N/A	N/A	N/A	N/A	N/A
DB00908	Quinidine	Sodium channel inhibitor	N/A	N/A	N/A	N/A	N/A
DB00215	Citalopram	SSRI	Active, not recruiting	Breast Cancer	N/A	N/A	https://clinicaltrials.gov/ct2/show/NCT00667121
DB00457	Prazosin	Statin	N/A	N/A	N/A	N/A	N/A
DB01076	Atorvastatin	Statin	Recruiting	Prostate Cancer	N/A	Phase III	https://clinicaltrials.gov/ct2/show/NCT04026230
			Recruiting	Breast Cancer	Metformin	Early Phase I	https://clinicaltrials.gov/ct2/show/NCT01980823
			Completed	Kidney Cancer	Zoledronate	Phase II	https://clinicaltrials.gov/ct2/show/NCT00490698
DB09241	Methylene blue	Treat methemoglobinemia	Completed	Sentinel Lymph Node (SLN) detection rate of Early Breast Cancer	N/A	N/A	https://clinicaltrials.gov/ct2/show/NCT02084784
DB06212	Tolvaptan	V2 antagonist	N/A	N/A	N/A	N/A	N/A

Ongoing and completed clinical trials that test the drug for therapeutic efficacy are listed. MOA, mechanism of action; N/A, not applicable.

## Repurposing of FDA Approved Drugs With p-gp Inhibitor Activity in Cancer Treatment

### Rationale

In light of previously reviewed clinical development history, small molecules specifically designed for p-gp inhibition were largely unsuccessful in clinical trials. Early generations of p-gp inhibitors were burdened with significant side effect toxicities, while newer generations of p-gp inhibitors generally had a more acceptable safety profile, yet still demonstrated limited efficacy.

We then sought to investigate current FDA approved drugs for their p-gp activity. We propose two rationales for this approach. First, FDA approval as well as real world experience indicates the safety profile of these drugs, therefore minimizing further development issues. Additionally, repurposing of these drugs in combination with current anti-cancer therapeutics may provide an additive effect to enhance efficacy.

### Discovery of Candidates for p-gp Inhibitor Repurposing

**Figure 1:**
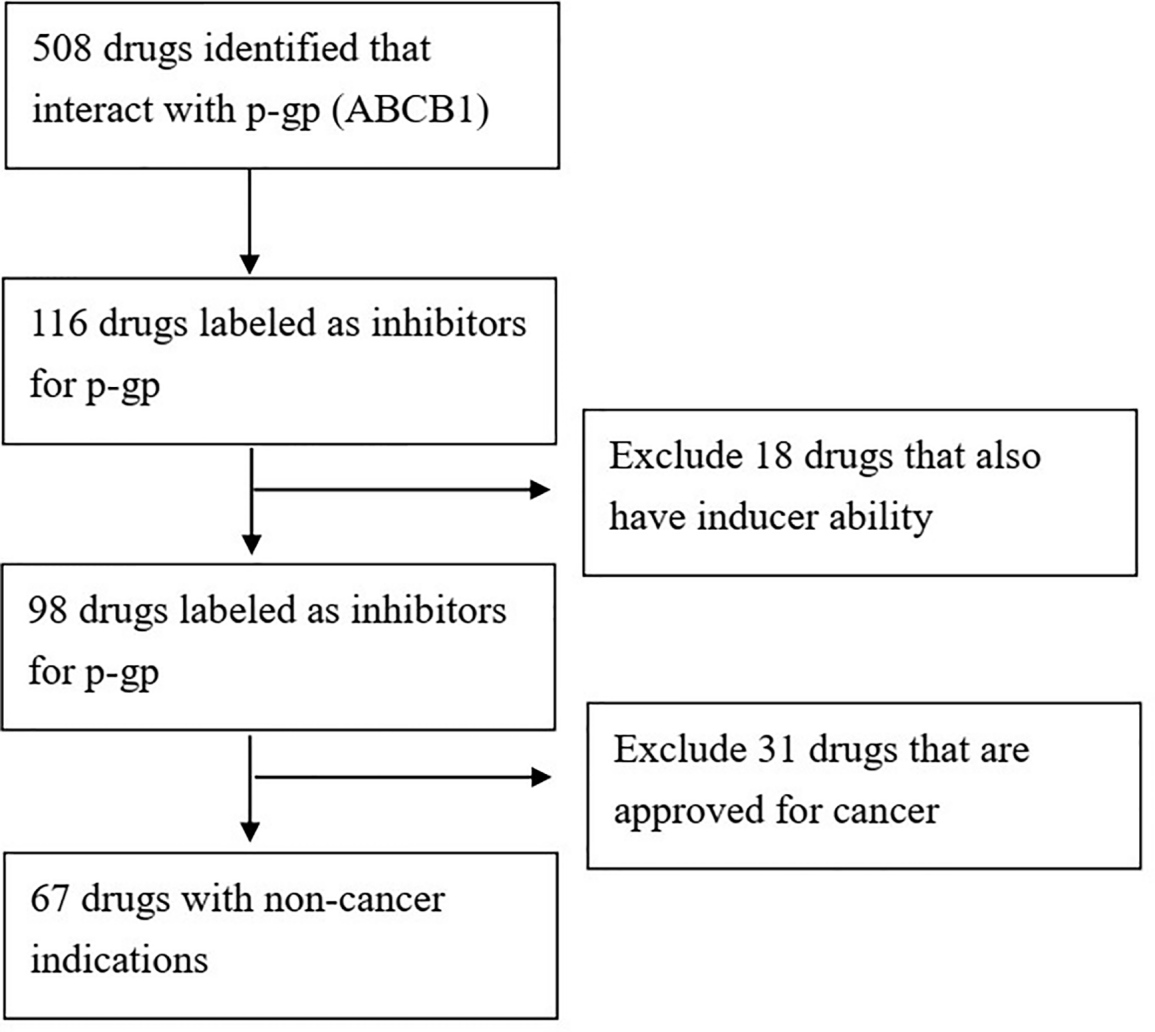
Flowchart of discovery of repurposing FDA approved drugs with p-gp inhibition abilities.

We queried the Drugbank database (https://www.drugbank.ca/) (54) for FDA approved drugs with demonstrated activity towards p-gp (gene symbol: *ABCB1*). The search resulted in 508 candidates. We then filtered out drugs without inhibitor activity for p-gp, leaving 116 drugs. We further filtered out drugs that had the “inducer” label, meaning that at least one reference reported upregulation of p-gp by this drug under specific circumstances. The final result was 98 FDA approved drugs with reported p-gp inhibition activity (**Figure 1**).

Interestingly, many drugs approved for anti-cancer properties were on this list of p-gp inhibitors. The known anticancer drugs included tyrosine kinase inhibitors (imatinib, sorafenib, dasatinib, gefitinib, nilotinib, erlotinib, and afatinib), PARP inhibitors (olaparib, rucaprib), CDK4/6 inhibitors (palbociclib, abemaciclib), taxanes (paclitaxel, cabazitaxel) and others. This finding leads to several observations: first, multiple agents with the same mechanism concomitantly inhibit p-gp suggest that this is a class effect, with a potential undiscovered mechanism. Secondly, the drugs of the same class that are not reported to possess p-gp inhibitor property (barring undiscovered p-gp inhibitor ability) may therefore possess a novel, unique chemical biological characteristic. For instance, two of the three approved CDK4/6 inhibitors (palbociclib, abemaciclib) inhibit p-gp, while ribociclib was not annotated with this property. Is the p-gp inhibition function not yet discovered in ribociclib, or does ribociclib possess a specific structural aspect that abolishes this possible ability? Third, these drug that intrinsically possess p-gp inhibitory functions suggest that additional p-gp inhibition with specific small molecules might provide limited additional benefit. This may be the case of taxanes, as several p-gp inhibitors were tested in combination with paclitaxel. It must be emphasized that the intrinsic p-gp inhibitory ability is uncharacterized for most of these drugs, so we cannot assess whether they possess strong p-gp inhibition function or otherwise.

We then excluded drugs that were approved for or possessed well established anti-tumor activities, filtering down the list to 67 agents. A summary of the final 67 drugs is shown in **Table 1**. Again, different agents of the same class appear, including proton pump inhibitors (Pantoprazole, omeprazole, lansoprazole, and esomeprazole), histamine blockers (ranitidine and loratadine), quinolones (levofloxacin and grepafloxacin), calcium channel blockers (amlodipine and nicardipine), phenothiazine (chlorpromazine) and others, hinting at possible class effect of p-gp inhibition. Interestingly, this feature may explain some previous observational studies. For instance, quinolones (ciprofloxacin or ofloxacin) when given in combination with doxorubicin as intravesicular treatment, significantly enhanced the cytotoxicity of doxorubicin towards cancer cells (55). We queried Clinicaltrials.gov database (https://clinicaltrials.gov/ct2/home) to search for ongoing or completed trials that used of these drugs with a cutoff date of July 2020. Specifically, we included the trials that investigate the therapeutic effect of these drugs in cancer, summarized in **Table 2**. In the following sections, we highlight some of the compounds that have been reported or investigated in combination with chemotherapy for anticancer treatment. We summarize the highlighted compounds in **Table 3**.

**Table 2:** Summary of major pg-1 inhibitors in clinical development.

Drug name	MOA	Phase	Adverse effects	Outcomes	References
First generation					
Verapamil	Calciumchannel blocker	phase II	Hypotension, cardiotoxicity	No clinical benefit	(11, 12)
Second generation					
Dexverapamil	calcium channel blocker	Phase II	Cardiotoxicity	ORR 9%, DCR 19% (18)	(18)
S9788	p-gp inhibitor	Phase I	arrhythmia	Further development limited by severe arrythmia	(20)
PSC-833	p-gp inhibitor	Phase III	NS	No benefit seen in phase III trials	(21–29)
Third generation					
Zosuquidar	p-gp inhibitor	Phase III	neurotoxicity	No clinical benefit seen in solid and hematological cancers	(31–37)
Elacridar	p-gp inhibitor	Phase I	neutropenia	Demonstrated increase in bioavailability of accompanying chemotherapy drug (oral topotecan, oral paclitaxel)	(38–44)
Tariquidar	p-gp inhibitor	Phase II	NS	Modest ORR in pretreated patients (5–10% in most trials) Demonstrated potential for development as radiotracer coupled with radioactive [11C] labeling	(45–53)

MOA, mechanism of action; ORR, Objective response rate; DCR, disease control rate (combination of response rate and stable disease rates); NS, Not significant.

**Table 3:** Summary of highlighted compounds that have been reported or investigated in combination with chemotherapy for anticancer treatment.

Compound name	Type	Approved clinical use	Clinical trials for anticancer treatment	Treatment regimen	Efficacy	Reference
Chlorpromazine	Phenothiazine	Nausea, vomiting, schizophrenia	N/A	N/A	N/A	N/A
Clarithromycin	Macrolide	Antibiotics	Lung cancer	Treosulfan pioglitazone Clarithromycin	Mild OS benefit (HR: 0.86)	(56)
Sildenafil	PDE inhibitor	Erectile dysfunction	Waldenstrom’s macroglobulinemia	Sildenafil	17 at least minor response	(57)

## Chlorpromazine

Chlorpromazine is a phenothiazine drug with p-gp inhibiting abilities. Clinically it possesses anti-emetic properties as well as antipsychotic properties. Interestingly, antipsychotics were another class of drug that were overrepresented in our p-gp inhibitor list (including paliperidone, haloperidol). Chlorpromazine is a p-gp inhibitor with an EC50 10 fold weaker than PSC833, but nevertheless is in low micromolar range (58). There has been much active research relating to chlorpromazine and its anticancer properties, as it has been reported to suppress the Hippo pathway related *YAP* signaling and cancer stemness (59), suppress chemoresistant cancer growth (60, 61), epigenetic anticancer mechanisms (62), and many other abilities. The ability to inhibit cancer stem cells seems to be a class effect of phenothiazines, and many novel derivatives are currently being developed towards this goal (63). It remains to be seen whether the ability to inhibit p-gp of phenothaizaines such as chlorpromazine will be a positive factor in enhancing antitumor activity either in monodrug use or in combination with other cytotoxic agents.

## Macrolides: Erythromycin, Azithromycin, and Clarithromycin

The FDA approved macrolide antibiotics (erythromycin, azithromycin, and clarithromycin) were all reported as p-gp inhibitors. This has been supported by many studies in the literature (64–66). Macrolides are well known CYP3A4 inducers (67, 68), thus it may interfere with pharmacokinetics with various results, since CYP3A4 and p-gp would both play a role in drug-drug interaction. Indeed, investigations regarding macrolides upon anticancer drugs have been made to further clarify whether efficacious drug effect can be adequately delivered (69). The macrolide clarithromycin has been studied in multiple clinical trials with cancer subjects, in combination with metronomic chemotherapy, abemaciclib, etc. (see **Tables 2**, **3**). In these trials, the rationale was mainly to study the CYP3A inhibitor activity of clarithromycin and its impact on the accompanying drug, but some have shown promising efficacy with possible potential for further follow up clinical trials.

## Phosphodiesterase Inhibitors: Sildenafil and Vardenafil

Two FDA approved phosphodiesterase inhibitors, sildenafil and vardenafil were reported to possess p-gp inhibition activities. Both sildenafil and vardenafil are potent PDE5 inhibitors that are widely used in erectile dysfunction. Interestingly, sildenafil has been actively studied for its role in anticancer treatment (70, 71), and has been proposed to potentiate cisplatin mediated tumor killing (71–73). While multiple mechanisms have been proposed for this phenomenon, the role of p-gp inhibition remain a very likely explanation. The anticancer roles of PDE5 inhibitors in single use and in combination with chemotherapy remains an active area of research and we anticipate more results to be reported on this topic.

## Conclusions

The clinical development of *bona fide* p-gp inhibitors has been rather disappointing in the past two decades. Although much progress has been made in medicinal chemistry to develop newer generation p-gp inhibitors, failure in clinical trials suggest that solely targeting p-gp may not be an efficacious strategy for cancer. A lot of the early generation p-gp inhibitors suffered from toxicity, while newer generation agents largely failed from lack of clinical response. A silver lining from these failed clinical trial is the development of the p-gp inhibitor tariquidar in nuclear tracer uptake enhancing agents for improvement in medical imaging. As we have demonstrated here, this is a promising field of research that may lead to clinical usefulness of p-gp inhibitors in the near future.

Repurposing current FDA approved drugs as a strategy for cancer development offers the benefit of well-known toxicity profile and mechanism of action, minimizing unexpected setbacks in clinical development. From the brief review of FDA approved drugs that possess p-gp inhibition activity, we highlight several candidates such as chlorpromazine and sildenafil that could have potential for further development, utilizing its role of p-gp inhibitor to enhance the potency of tumor cytotoxic agents. As highlighted in **Table 2**, many of these drugs have underwent clinical trials, either as monotherapy or in combination for cancer patients. Repurposing “old” currently common drugs for cancer treatment is an area of heightened interest, and many drugs with long history of clinical use have been discovered for novel mechanisms. One example is metformin (FDA approved in 1998), which has recently been reported to have promising roles for anticancer therapies in both preclinical and clinical studies. Metformin is now being investigated in numerous active clinical trials for discovering a positive role in cancer. We are hopeful that future research would define a role of repurposed p-gp inhibitors in cancer treatment and in improving patient survival.

## Author Contributions 

J-IL performed the literature search and drafted the manuscript. Y-JT and M-HC performed the data curation and construction of the Tables. C-YH contributed to study design, insight, and manuscript drafting. PC designed the study, assisted in the literature search, revised and refined the completed manuscript and the Figure and Tables. All authors contributed to the article and approved the submitted version.

## Funding

This study was partially funded by the grants: V109C-009 and MOST-109-2314-B-075-080 (to PC), and 108DHA0100627 (to PC, J-IL).

## Conflict of Interest

The authors declare that the research was conducted in the absence of any commercial or financial relationships that could be construed as a potential conflict of interest.
